# An orthogonal approach for analysis of underivatized steroid hormones using ultrahigh performance supercritical fluid chromatography-mass spectrometry (UHPSFC-MS)

**DOI:** 10.1007/s00702-024-02862-3

**Published:** 2024-11-15

**Authors:** Perry Devo, Victoria Cretu, Harsha Radhakrishnan, Darren Hamilton-Pink, Stergios Boussios, Saak V. Ovsepian

**Affiliations:** 1 Faculty of Engineering and Science, University of Greenwich London, Chatham Maritime, London, ME4 4TB UK; 2 Department of Natural Sciences, Middlesex University, London, NW4 4BT UK; 3 Department of Medical Oncology, Medway NHS Foundation Trust, Gillingham, ME7 5NY UK; 4 Faculty of Medicine, Health, and Social Care, Canterbury Christ Church University, Canterbury, CT2 7PB UK; 5 Faculty of Life Sciences & Medicine, School of Cancer & Pharmaceutical Sciences, King’s College London, Strand, London, WC2R 2LS UK; 6 Kent Medway Medical School, University of Kent, Canterbury, CT2 7LX UK; 7 AELIA Organization, 9th Km Thessaloniki-Thermi, Thessaloniki, 57001 Greece

**Keywords:** Steroid hormones, UHPLC-MS, Solid phase extraction, Supercritical fluid, Biomarkers, UHPSFC-MS

## Abstract

The crucial role of steroid hormones in health and diseases merits their high-throughput, accurate and affordable measurements in biological specimens. Despite advances in analytical methods, sensing and quantifying steroid hormones remains challenging. Immunoassays offer excellent sensitivity but are inherently labour-intensive, costly, and prone to false positives. Mass spectrometry (MS) has been increasingly utilised, with the main hurdle being the isobaric tendencies of similar analytes, which complicates their separation and accurate quantification. This study compares ultrahigh-performance supercritical fluid chromatography separation (UHPSFC) and ultra-high-performance liquid chromatography (UHPLC) for MS detection. It optimises the column chemistry, temperature, and pressure to provide an operational protocol for the resolution and quantification of analytes. It presents the systematic characterisation of UHPSFC-MS performance by investigating spiked blood samples using Solid-Phase Extraction (SPE) and describes the matrix effects associated with MS measurements. Although both separation methods showed adequate resolution, specificity, and retention time, UHPSFC-MS was superior for five out of seven columns tested. With added high-throughput capacities, UHPSFC-MS, thus, offers an optimal solution for the analysis of steroid hormones for research, medical chemistry, and clinical diagnostics.

## Introduction

Steroid hormones are small biomolecules with a wide range of signalling roles, including generation and control of stress response, modulation of immune activity and brain mechanisms, and regulation of reproductive functions. The non-polar nature and lipid solubility render them penetrable across biological barriers, acting in addition to surface receptors at the genome level (Norman et al. 2004; Wilkinson and Brown 2015). Produced and released by specialised organs and tissues, their activity is tightly regulated and can be set off the balance by multiple disease conditions (Barton et al. 2021). Corticosteroids, for instance, play a crucial role in gauging the response to various stressors, with their long-term increase in circulation impairing glucose metabolism and causing immunosuppression (Bereshchenko et al. 2018; Lee et al. 2015). Gonad steroids control reproductive functions and contribute to the onset of post-partum and menopause-related depression, as well as postmenopausal cognitive decline and memory loss in Alzheimer’s disease (Noyola-Martinez et al. 2019; Studd and Nappi 2012; Zhang et al. 2023). Altered production and activity of gonad hormones have been recently implicated also in the pathobiology of cancer (Dahmani et al. 2023).

The importance of steroids in physiological and diseased states warrants quantification and control of their effects. While offering excellent sensitivity, immunoassays are inherently labour-intensive, costly, and prone to cross-reactivity and false positives (Holst et al. 2004; Karashima and Osaka 2022). The readouts of immunoassays can also be biased by autoimmune diseases and immunotherapy (Dasgupta 2016; Castro and Gourley 2010). Mass-spectrometry (MS) has been increasingly used for quantifying steroids. The process involves isolating individual hormones before measurements. Although ultra-high performance liquid chromatography (UHPLC) and gas chromatography (GC) have been extensively utilised, achieving a clear separation for related substances has remained challenging (Gupta et al. 2022; Schurig 1988; Atapattu and Temerdashev 2023). By improving the sample preparation process, the total analysis time can be reduced, albeit with the drawbacks of higher isobaric interferences of structurally similar steroids, which can lead to ion suppression and loss of sensitivity (Temerdashev et al. 2022).

Ultra-high performance supercritical fluid chromatography (UHPSFC), which uses supercritical fluid as a mobile phase, partly overcomes the challenge of isobaric interferences. It applies separated analytes into the solvent stream, where the isolated components are de-solvated and re-dissolved in an isocratic diluent (Losacco et al. 2021; Neumann et al. 2022). This protocol enables the entry of a more concentrated sample into the MS source. While the processes behind changes in retention factors with column temperature and pressure variations require elucidation, evidence suggests that the pressure effects are contributed by mobile phase density. The impact of varying column temperatures is understood even less, with reduced mobile phase density viewed as specific to the used analytes (Si-Hung and Bamba 2021).

In this study, we examine if UHPSFC-MS can quantify steroid hormones in biological specimens without derivatisation. We show that UHPSFC-MS is capable of rapid and reliable measurements of nine common steroids in solvents and horse blood without derivatisation, with excellent resolution and specificity. The favourable outcome of our measurements casts a promising outlook for applying the described herein approach in translational research, medical and analytical chemistry, and diagnostics.

## Materials and methods

Nine most common steroid hormones, progesterone, hydrocortisone, corticosterone, estriol, aldosterone, allopregnanolone, β-estradiol, estrone and testosterone, were purchased from Fisher Scientific (> 97% purity, Fisher Scientific, UK). Defibrinated horse blood pooled from males and females was purchased from Fisher Scientific (Thermo Scientific Oxoid, SR0050C, UK). A total of 9 column chemistries were investigated: two UHPLC and seven UHPSFC (Waters, Massachusetts, USA). Unless otherwise stated, all columns were 100 mm long with a 1.7 µM particle size. UHPLC ACQUITY BEH column stationary phases were C18 and HSST3, with an internal diameter of 2.1 mm. UHPSFC Torus columns used were fluorophenyl (FP), high-strength silica C18 (HSS SB), diol (Diol), diethylamine (DEA), 1-amino anthracene (1-AA), 2-picolyl (2-PIC) and cellulose-1 (CEL-1) which had a 2.5 µM particle size, a chiral sorbent, with an internal diameter of 3.0 mm. Liquid CO_2_ was purchased from BOC (CP grade, > 99.9%), while eluents (methanol and water) and additives (formic acid and ammonium hydroxide) were purchased from Fisher Scientific (MS grade). Buffers added to mobile phase eluents were formic acid and ammonium hydroxide (0.1% v/v). UHPSFC separation was carried out on an ACQUITY UPC^2^ (Waters, Massachusetts, USA) fitted with an 8-column oven. UHPLC was separated on an ACQUITY I-class UHPLC (Waters, Massachusetts, USA). All analysis was carried out on a Qda mass detector (Waters, Massachusetts, USA) with a selected ion recording (SIR) set to detect the mass of each analyte (Table 1).

**Table 1:** Summary of the mass-spectrometry detection parameters for the investigated nine selected steroid hormones

Hormone	Chemical formula	Mass (g/mol)	SIR channel	Adduct	Ionization mode	LOD (µg/mL)	LOQ (µg/mL)
Progesterone	C_21_H_30_O_2_	314.2	315.2	[M + H]^+^	ES + ve	0.016	0.051
Testosterone	C_19_H_28_O_2_	288.2	289.2	[M + H]^+^	ES + ve	0.021	0.070
Hydrocortisone	C_21_H_30_O_5_	362.2	363.2	[M + H]^+^	ES + ve	0.004	0.012
Aldosterone	C_21_H_28_O_5_	360.2	361.2	[M + H]^+^	ES + ve	0.066	0.219
Corticosterone	C_21_H_30_O_4_	346.2	347.2	[M + H]^+^	ES + ve	0.046	0.138
Estrone	C_18_H_22_O_2_	270.2	269.2	[M-H]^-^	ES -ve	0.062	0.217
β-Estradiol	C_18_H_24_O_2_	272.2	271.2	[M-H]^-^	ES -ve	0.073	0.234
Estriol	C_18_H_24_O_3_	288.2	287.2	[M-H]^-^	ES -ve	0.091	0.283
Allopregnanolone	C_21_H_34_O_2_	318.3	301.3	[M + H-H_2_ O]^+^	ES + ve	0.021	0.060

We used horse blood as a complex matrix to verify the utility of the UHPSFC-MS for quantifying steroid hormones. The matrix effects experienced when recovering hormones from the horse blood were quantified by undertaking two measurements: (1) recovery of a known spiked solution of horse blood, “*A*_*spiked blood*_*”*, and (2) recovery using hormones dissolved in water: methanol (H_2_O: CH_3_OH, 95:5) solution as a control. The control recovery was tested using a stock solution (1 µg/mL) of analytes in H_2_O: CH_3_OH (95:5). Extracts were quantified by deriving the linear equation using a regression approach. The correlation of each linear regression was r^2^ > 0.99. Concentrations were then classified as “*A*_*solventsolution*_” and used to calculate the matrix effects.

The general procedure for extracting steroid hormones from horse blood is as follows. Blood (1 mL) was spiked with a solution containing nine steroid analytes to afford a final known concentration of 0.6 µg/mL. The solution was then vortexed for 30 s, at which point acetonitrile (1 mL) containing either 0.1% (v/v) ammonium hydroxide, 0.1% (v/v) formic acid or no additive was added along with brine (250 µL) and magnesium sulphate (50 mg). The solution was centrifuged for 10 min at 14,000 rpm (Wozniak et al. 2019). An aliquot (600 µL) was then removed and subjected to SPE separation using either a C18 or PSA sorbent according to the protocol reported by Pouech and co-workers (Pouech et al. 2012). All measurements were conducted in triplicate, with statistical analysis and tests carried out using Microsoft Excel. One-way ANOVA was used to compare various datasets, while for paired comparison, we used paired Student’s *t*-test, with *p* < 0.05 taken as a significant difference between sets of measurements. All graphs and histograms are plotted in Excel, and the results are presented using Adobe Illustrator (CS6).

The matrix effects (ME) were calculated according to the following Eq. 1:

$$\:ME\:\left(\%\right)=\left(\frac{{A}_{spiked\:blood}}{{A}_{solvent\:solution}}-1\right)\times\:100$$

The sample variations and accuracy of readouts were quantified based on calculating relative standard deviation (RSD) using the following Eq. 2:

$$\:RSD\:\left(\%\right)=\frac{standard\:deviation\:recovery}{mean\:recovery}\times\:100$$

## Results

First, we quantified the effects of varying column pH on the retention time of analytes in UHPLC and UHPSFC columns. The initial MS screening was performed using two UHPLC and seven UHPSFC columns. Table 2 summarises the results obtained, showing the impact of column phase, sorbent, and pH variations on the retention time.

**Table 2:** Summary data showing differences in retention time when using nine different columns (including two different pH for UHPLC). General conditions for UHPSFC are as follows: 0 min (CO2:MeOH, 95:5), 3.75 min (CO2:MeOH, 50:50), 4.00 min (CO2:MeOH, 50:50), 4.10 min (CO2:MeOH, 95:5) and 5.00 min (CO2:MeOH, 95:5) with a flow rate of 1 mL/min, ABPR pressure of 2000 and column temperature of 40 ºC. General conditions for UHPLC separation are as follows: 0 min (H2O: MeOH, 95:5), 0.2 min (H2O: MeOH, 95:5), 3.5 min (H2O: MeOH, 5:95), 4.0 min (H2O: MeOH, 5:95), 4.1 min (H2O: MeOH, 95:5), 5 min (H2O: MeOH, 95:5) with an additive concentration of 0.1% v/v. analyses were carried out in triplicate to ensure repeatability (*N* = 3)

Hormone	UHPLC-MS				UHPSFC-MS						
	C18 (FA)	C18 (NH3OH)	HSST3 (FA)	HSST3 (NH3OH)	FP	HSS SB	Diol	DEA	1-AA	2-PIC	Cel-1
Progesterone	3.07	3.17	3.32	3.32	1.57	2.19	1.19	0.96	2.89	1.18	3.25
Testosterone	2.52	2.17	2.78	2.78	1.99	2.57	2.25	1.90	3.05	2.21	3.37
Hydrocortisone	1.99	2.66	2.31	2.21	2.42	2.77	3.63	2.92	3.47	3.01	3.77
Aldosterone	1.87	2.04	2.19	2.09	2.40	2.84	2.83	2.65	3.72	2.78	3.95
Corticosterone	2.25	2.4	2.48	2.47	2.29	2.74	2.67	2.56	3.70	2.67	4.03
Estrone	2.60	2.75	2.89	2.81	1.70	2.32	2.42	2.49	3.02	2.56	3.63
β-Estradiol	2.44	2.59	2.75	2.70	2.14	2.32	2.87	2.93	3.37	3.01	3.87
Estriol	1.82	1.99	2.18	2.06	2.52	2.87	3.31	3.36	3.65	3.40	3.80
Allopregnanolone	3.30	3.38	3.34	3.34	1.22	2.42	1.83	1.46	2.71	1.87	3.41

**Fig. 1:**
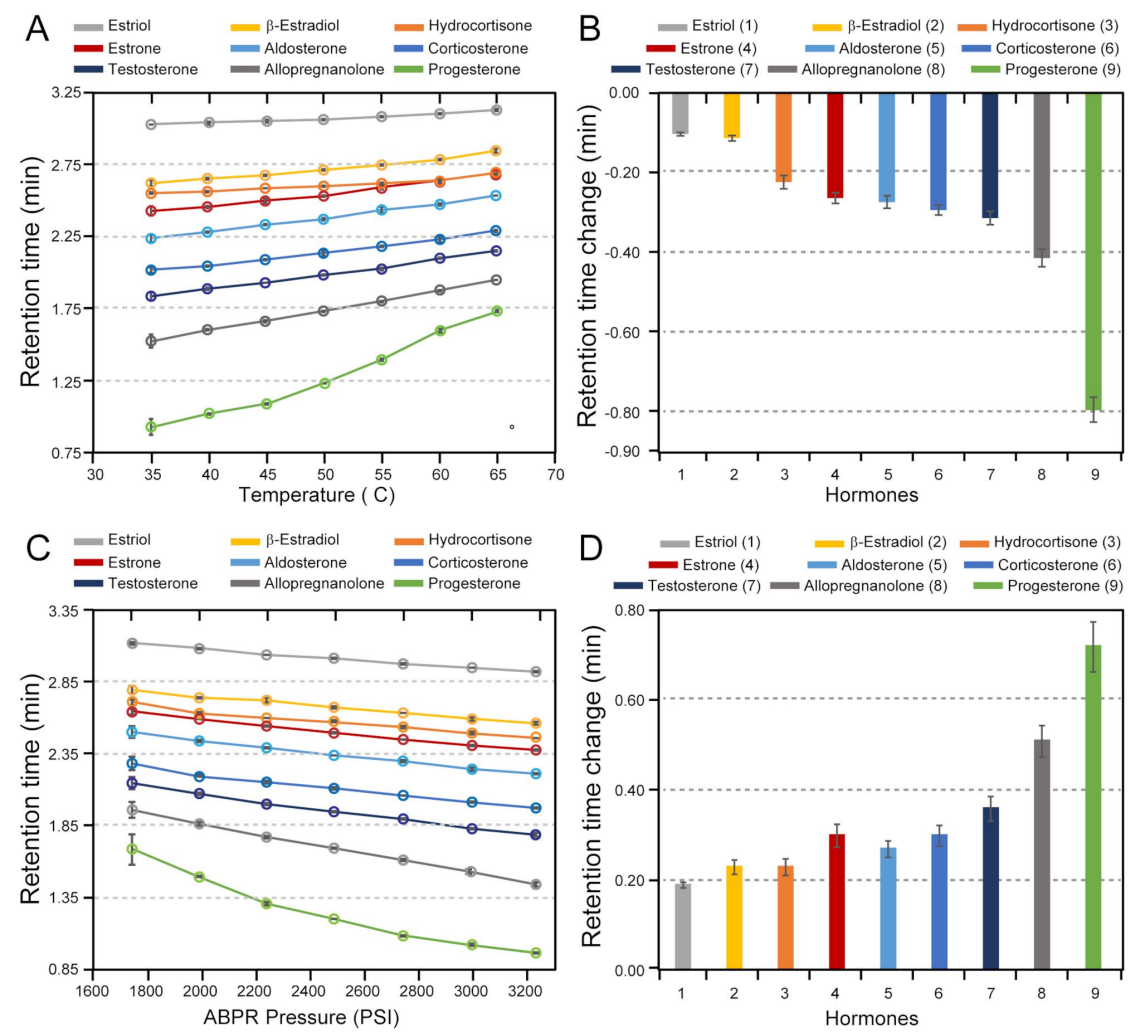
Summary of the effects of the temperature and pressure on measurements of nine underivatized common steroid hormones using UHPSFC method. (**A**) Summary graph of retention-temperature relation showing the effect of increasing column temperature on the steroid analyte retention time. (**B**) A bar graph demonstrating the retention-time distribution of each analyte between 35 and 65 ºC. (**C**) A summary graph of the retention-pressure relation shows the effect of increasing ABPR pressure on the retention time of all the steroid analytes. (**D**) A bar graph illustrating the retention time distribution between 1850 and 3250 psi. All data points are mean averages of measurements carried out in triplicate (*N* = 3)

In UHPLC experiments, an increase in the pH of the mobile phase reduced the retention time, and effect that was more pronounced using a C18 sorbent (paired Student *t*-test, *p* < 0.05 for all hormones). Several analytes showed significant co-elution through UHPLC separation. Specifically, estriol and aldosterone consistently displayed co-elution tendencies in sorbents with high and low pH. Allopregnanolone and progesterone co-eluted in the HSST3 sorbent. In the case of UHPSFC, the choice of sorbents with differing polarities was pivotal in achieving optimal separation. Sorbents with higher polarity demonstrated superior separation capabilities to the relatively less polar FP and HSS SB sorbents, with the diol sorbent achieving optimal peak resolution. To improve peak resolution further, we explored the effects of varying column temperature (35 to 65 ºC, 5 ºC increments) and ABPR pressure (1750 to 3250 psi, 250 psi increments). Figure 1 presents a graphical summary of the results of UHPAFC experiments.

The results of our measurements show a consistent trend in retention time response across analytes when varying temperature and pressure, which agrees with findings reported by Wang and Ovchinnikov (Ovchinnikov et al. 2020; Wang et al. 2011). Increasing column temperature caused an increase in the retention for all analytes (Fig. 1A), while increasing column pressure decreased their retention times (Fig. 1C**).** The retention time changes for individual analytes were non-uniform when column temperature and pressure were modified. Concerning column temperature, earlier eluting analytes show a more pronounced change in retention time compared to later eluting analytes. Figure 1B and **D** summarises changes in retention time as a function of temperature and pressure, showing the difference between 35 and 65 º C and 1750 and 3250 psi respectively. Compared to effects of temperature variations, in pressure variation experiments, earlier eluting analytes displayed less variation in retention time, while analytes eluting later showed a more change in retention time (Fig. 1B, D).

**Fig. 2:**
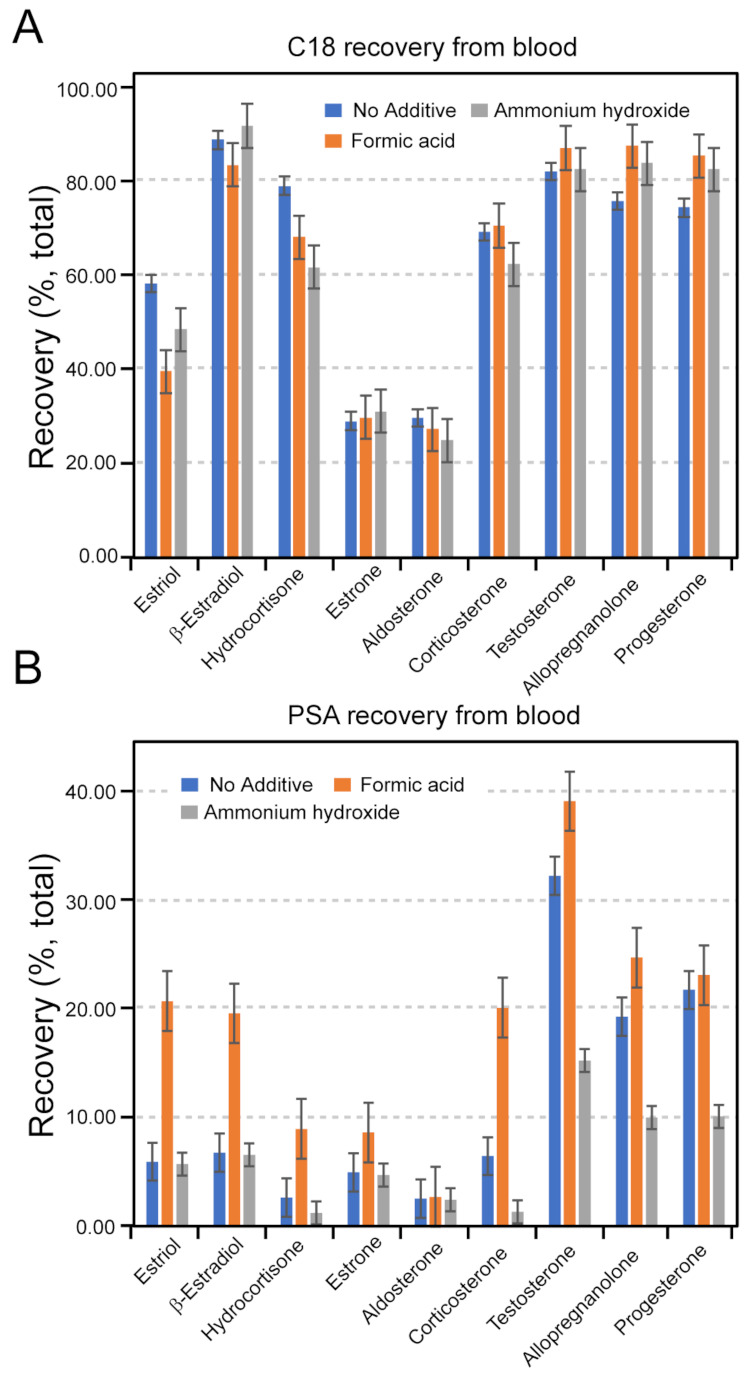
Summary bar graphs of the values of recovery of a 1 µg/mL hormone solution doped into whole blood retrieved using a C18 SPE (**A**) and PSA SPE (**B**) sorbents with the addition of (0.1% v/v) formic acid or ammonium hydroxide in the extraction solvent. All data points are mean averages of measurements carried out in triplicate (*N* = 3)

Next, we tested the utility of UHPSFC for the detection of steroids in clinical chemistry and diagnostics by recovering nine steroid hormones from the whole blood of a horse, as specified in Materials and Methods. For these experiments, column temperature and ABPR pressure were set to 50 º C and 2000 psi, respectively. First, a solution of hormones was recovered from C18 and PSA SPE sorbents to identify the maximum recovery from an uncomplex sample matrix. A stock solution of the nine steroid hormones was then spiked into whole horse blood for a final concentration of 0.6 µg/mL. We chose this concentration as it fell within the limits of reliable detection and was above the serum concentration naturally occurring in the sample (Caprioli et al. 2021; Genangeli et al. 2017). The blood sample was subsequently analysed with concentrations determined for each hormone. The results are summarised in Fig. 2A **and B**, which show the recovery of each analyte, using C18 and PSA sorbents, under low (3.0), neutral (7.0) and high (12.0) pH.

As clearly visible from these studies, the recovery across all nine analytes was more effective using C18 sorbent than that of PSA. Indeed, the recovery using C18 sorbent showed excellent selectivity towards steroid hormones, particularly towards β-estradiol, testosterone, allopregnanolone and progesterone, with values exceeding 75%. When buffering the pH of the extraction solvent for the C18 sorbent, which can vary the selectivity on the stationary phase, the differences were negligible (ANOVA, *p* > 0.05 for all hormones). Comparison between additives, however, showed a significant recovery increase when including 0.1% formic acid in the extraction solvent using PSA as a sorbent (ANOVA and paired Student *t*-test, *p* < 0.05 for all hormones). The differential effects likely result from the generation of positive ions during the work-up procedure, leading to a stronger analyte sorbent interaction (Marwah et al. 2020). It is known that matrix effects can significantly impact the accuracy and reliability of analytical measurements during SPE (Mirmont et al. 2022; Andrew and Homer 2016, 2020). Therefore, their accurate quantification and considerations are essential for obtaining reliable results that reflect the concentration of analytes within a sample. Furthermore, RSD calculation is a crucial metric for evaluating precision in SPE. Matrix effects and precision of each method for each hormone under a given condition were calculated using Eqs. 1 and 2. Table 3 summarises the impact of extracting steroid hormones from horse blood in three individual samples, demonstrating high accuracy and reproducibility (variance < 10%). Notably, there was considerable variability of matrix effects between different analytes, which is likely to result from changes in the ionisation of the analytes in co-eluting components.

**Table 3:** Matrix effects (ME, %), relative standard deviations (RSD, %) and calibration coefficient of recovered analytes using pH-buffered blood plasma. All data points are averages from three replicates (*N* = 3)

Sorbent chemistry	C18						PSA						
Additive	No Additive		FA		NH4OH		No Additive		FA		NH4OH		
Hormone	ME	RSD	ME	RSD	ME	RSD	ME	RSD	ME	RSD	ME	RSD	Coefficient
Progesterone	1.44	1.61	16.35	6.97	12.49	3.82	-4.23	6.17	1.81	4.32	-55.29	6.35	0.9966
Testosterone	-20.83	1.74	-15.98	6.47	-20.40	8.16	37.83	1.92	67.06	3.79	-34.68	8.85	0.9959
Hydrocortisone	101.85	4.62	73.99	6.63	57.90	7.13	-70.34	6.37	-1.55	1.49	-86.00	6.35	0.9942
Aldosterone	-48.73	5.14	-48.73	7.39	-56.81	8.57	-55.55	0.30	-52.61	4.31	-57.37	0.08	0.9905
Corticosterone	108.99	1.11	113.04	6.89	88.52	9.27	-48.88	9.13	58.12	4.19	-89.10	7.18	0.9911
Estrone	-38.16	1.41	-36.48	4.71	-33.82	9.30	-68.86	4.84	-46.26	5.20	-70.54	4.95	0.9942
β-Estradiol	37.10	5.42	28.95	5.94	41.69	7.47	-70.21	7.79	-14.50	9.54	-71.14	9.28	0.9908
Estriol	56.02	4.54	-70.08	7.82	-63.38	3.83	-45.59	5.93	88.18	4.14	-47.76	4.73	0.9918
Allopregnanolone	43.15	0.90	65.24	9.13	58.21	2.98	-10.48	2.22	14.63	3.74	-53.48	3.76	0.9963

## Discussion

Experimental and clinical studies investigating steroid hormones have measured changes in their concentration and activity for several decades. Although traditionally, individual steroids have been analysed, the investigation of panels of steroid hormones and their higher-level interactions is gaining widespread traction due to the diagnostic relevance of combined changes and the arrival of technologies capable of high-throughput measurements and analysis of large volumes of data (Schiffer et al. 2019; Duskova et al. 2020; Andrew and Homer 2016).

Kock and co-workers have shown the utility of UHPSFC for orthogonal separation of isobaric steroid hormones after derivatisation (de Kock et al. 2018). In this study, we examine for the first time, whether UHPSFC-MS can quantify steroid hormones in biological specimens without derivatisation. We show that UHPSFC-MS enables rapid recovery and measurements of nine prevalent steroid hormones in solvents and horse blood without derivatisation, with excellent resolution and specificity. The reassuring outcome of our measurements casts a promising outlook for applying described herein approach in biomedical research, analytical and medical chemistry, and diagnostics. The results of separating nine underivatized steroid hormones using UHPSFC-MS and comparison with those from UHPLC-MS show that while both methods have been highly instructive in resolution, specificity, and retention time, UHPSFC-MS outperformed UHPLC-MS in 5 out of 7 columns tested. It is noteworthy that common MS additives such as formic acid and ammonium hydroxide had negligible effects on hormone recovery when used in a C18 sorbent, whereas in PSA stationary phase, the recovery of hormones was higher in formic acid. Several studies showed that changes in retention time can vary depending on the analyte stationary phase interaction (Tarafder et al. 2015; Ovchinnikov et al. 2022).

**Fig. 3:**
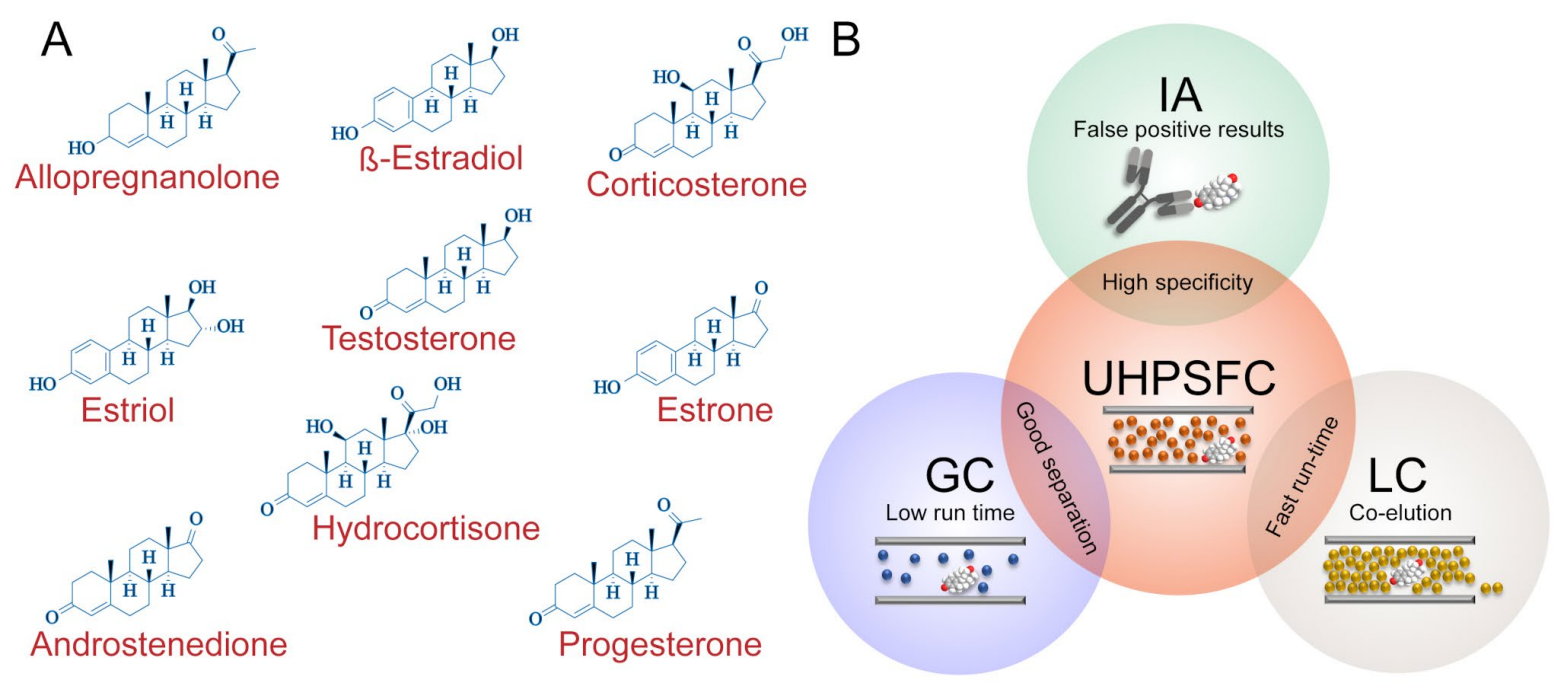
(**A**) Representation of the chemical structure of nine analysed steroid hormones using UHPSFC in this study. (**B**) Venn diagram demonstrating the shared advantage of the UHPSFC with immunological assays (IS), gas chromatography (GS) and liquid chromatography (LC) for orthogonal analysis of underivatized steroid hormones

Although we observed measurable matrix effects in both, UHPLC-MS and UHPSFC-MS experiments, they did not demote the overall performance and consistency of measurements across the spectrum of steroids. The overall outcome of our experiments suggests that as an analytical approach, UHPSFC-MS combines advantages of three main methodologies for quantifying steroid hormones, i.e. as high-specificity of immunological assays (IA), good separation of gas chromatography (GS) and fast run-time of liquid chromatography (LC) (Fig. 3). The results of our analysis deem UHPSFC-MS as a method of choice for orthogonal measurements of panels of underivatized steroids in biological specimens, supporting its applications for high-throughput clinical and diagnostic studies.

## Abbreviations

MS: Mass spectrometry
UHPLC: Ultrahigh Performance Liquid Chromatography
UHPSFC: Ultrahigh Performance Supercritical Fluid Chromatography
ABPR: Automated back pressure regulator
SIR: Selected ion recording
SPE: Solid Phase Extraction
FP: Fluorophenyl
HSS SB: High Strength Silica C18
DEA: Diethylamine
1-AA: 1–aminoanthracene
2-PIC: 2–Picoloyl
CEL-1: Cellulose–1
PSA: Primary Secondary Amine
ME: Matrix Effects
RSD: Relative Standard Deviation
